# Emerging Vaccine Technologies

**DOI:** 10.3390/vaccines3020429

**Published:** 2015-05-26

**Authors:** Rebecca J. Loomis, Philip R. Johnson

**Affiliations:** The Children’s Hospital of Philadelphia Research Institute, Philadelphia, PA 19104, USA; E-Mail: loomisr@email.chop.edu

**Keywords:** vaccine, reverse vaccinology, structural vaccinology, epitope-scaffold, immunoprophylaxis

## Abstract

Vaccination has proven to be an invaluable means of preventing infectious diseases by reducing both incidence of disease and mortality. However, vaccines have not been effectively developed for many diseases including HIV-1, hepatitis C virus (HCV), tuberculosis and malaria, among others. The emergence of new technologies with a growing understanding of host-pathogen interactions and immunity may lead to efficacious vaccines against pathogens, previously thought impossible.

## 1. Introduction

History has shown that successful vaccination not only prevents incidence of disease but also has contributed to significant and dramatic improvements in public health worldwide. Despite these triumphs, many infections are not preventable with current vaccine strategies or technologies. These infections represent a major cause of mortality worldwide. For many pathogens, natural infection leads to immunity protecting the host from re-infection. Many highly successful vaccines, such as live-attenuated or inactivated vaccines, rely on direct mimicry of natural immunity induced by the pathogen. However, vaccines have not been developed against certain infections that either fail to induce sterilizing immunity following natural infection (*i.e*., respiratory syncytial virus (RSV), malaria), those that lead to persistent or latent infection (*i.e*., HIV-1, hepatitis C virus (HCV)) or those with high degrees of variability (*i.e*., dengue, HIV-1). This suggests that a viable vaccine would need to induce a protective immune response beyond the mechanisms evolved by nature. As new technologies emerge and our understanding of host-pathogen interactions and immunity grows, development of vaccines against impervious pathogens, using only the components of the pathogen or elements of the immune response necessary to elicit a protective immune response, may become possible.

## 2. The First Vaccines

Vaccination was designed to protect individuals from an infectious agent by injecting a weakened or attenuated form of the infectious pathogen, leading to immunity from infection of the injected pathogen in the individual. In 1796, Edward Jenner made a landmark discovery, giving rise to one of the first vaccines in the Western World. He observed that milkmaids were generally immune to smallpox and hypothesized that the pus in the blisters milkmaids received from cowpox, a disease similar to smallpox but less virulent, provided protection from smallpox. Upon testing, Jenner demonstrated this to be true [1]. Smallpox, a severe human disease, was eradicated through a global vaccination campaign in 1979.

Louis Pasteur continued to refine Jenner’s work using weakened forms of a pathogen to promote immunity to a virulent form. Pasteur “artificially” attenuated pathogens for use as vaccines (*i.e*., anthrax (oxidizing potassium dichromate), chicken cholera (spoiled bacteria), rabies (dried affected nerve tissue)). Thus, in these cases, a naturally weakened form of the disease pathogen did not need to be identified. Pasteur’s principles of vaccination—isolate, inactivate and inject—form the basis for many of the first vaccines [2,3]. Improvements and innovation in mammalian cell culture technology in the second half of the twentieth century led to the propagation of pathogens *in vitro* and development of live-attenuated vaccines against polio, measles, mumps, rubella and varicella. For example, the component viral strains of the MMR (measles, mumps, rubella) vaccine were developed in either embryonated hens’ eggs or chick embryo cell cultures (measles and mumps) and human WI-38 cells derived from embryonic lung tissue (rubella) to allow for production of more virus and attenuation through growth and passage in non-human cells or cell culture.

The first century of vaccine development was based on using the entire disease-causing pathogen in a killed or live-attenuated form (artificially or through *in vitro* cell culture propagation) that did not lead to clinical infection but induced protective immunity. These are often termed conventional/traditional vaccines. In developed countries, national immunization programs have drastically reduced many of the viral and bacterial infections that traditionally affected children (*i.e*., measles, mumps, rubella, diphtheria, tetanus, pertussis). Inactivation and attenuation of the pathogen were the first choice for vaccine development for many years. Difficulty in cultivating some pathogens *in vitro* and the fact that even an attenuated pathogen may result in detrimental or unwanted immune responses showed that these approaches were impractical for pathogens that, for example, exhibit antigenic hypervariability (HIV-1, HCV), exacerbate disease (RSV, dengue) [4,5] or have an intracellular phase (tuberculosis, malaria) [6].

## 3. The Second Renaissance of Vaccine Development

Due to the limitations of traditional/conventional vaccine approaches against more difficult diseases, development of new methods was necessary. Subunit vaccines offer the potential to develop safe and highly characterized vaccines that direct immune responses toward specific pathogenic determinants. Subunit vaccines use only part of a target pathogen (*i.e*., protein/peptide, carbohydrate antigens, not the whole pathogen) to induce an appropriate response from the immune system. The targeted subunit is generally abundant and conserved in the pathogen, visible to the immune system and able to elicit a protective immune response. Immune responses can be refined through optimization of delivery systems, tuning the size of particulate vaccines, targeting specific immune cells and adding components to aid vaccine efficacy (adjuvants).

There are two ways to construct a subunit vaccine: (1) by isolating a specific protein from the pathogen and presenting it as an antigen on its own (*i.e*., protein) or (2) by genetic engineering (*i.e*., expressing an antigen in a vector). (1) Protein-based subunit vaccines present an antigen to immune system without viral particles, using a specific, isolated protein from the pathogen. A weakness of the technique is that isolated proteins, if denatured or having other subtle structural changes, may result in binding to different antibodies than would be recognized by the native protein of the pathogen. Acellular pertussis contains inactivated pertussis toxin (protein) and may contain one or more bacterial components (*i.e*., filamentous hemagglutinin, pertactin, fimbriae). Pertussis toxin is detoxified either by treatment with a chemical (*i.e*., hydrogen peroxide, formalin, glutaraldehyde) or by using molecular genetic techniques [7,8]. Once injected, the inactivated toxins (toxoids) elicit an immune response against the toxins, but, unlike the toxins, do not cause disease. “Acellular” pertussis contains less endotoxin than the “whole cell” pertussis vaccine [9,10], yielding lower rates of both mild and severe side effects [8], however, an increase in safety resulted in decreased vaccine effectiveness. (2) The second method, genetic engineering, involves cloning an antigen gene from the targeted virus/bacterium into another virus (viral vector), yeast or attenuated bacterium to make a recombinant virus/bacterium. The HepB vaccine contains one of the viral envelope proteins, hepatitis B surface antigen (HBsAg), produced by yeast cells into which the genetic code for HBsAg has been inserted. When the carrier virus/bacterium reproduces or the producer cell metabolizes, the vaccine protein is also created. Another example is the bivalent or quadrivalent HPV vaccine that is composed primarily of virus-like particles (VLPs) that self-assemble from copies of L1, the major structural protein of the HPV virion. These VLPs are free of DNA and are non-infectious. The bivalent HPV vaccine is comprised of two L1 proteins that were cloned into the baculovirus expression vector, expressed in insect cells, purified and formulated with adjuvant. The quadrivalent HPV vaccine is comprised of four L1 antigens produced from the yeast *Saccharomyces cerevisiae* expression system generating VLPs, which are then formulated with adjuvant. The end result of both approaches is a recombinant vaccine. Following injection, the immune system recognizes the proteins expressed in the vaccine as foreign, an immune response is mounted, providing future protection against the target pathogen.

The development of subunit vaccines was significantly aided by the advent of rDNA technology where recombinant viral genomes were rapidly exploited as gene carriers. Viral vectors have many attractive features including ease of construction and straightforward production of virus stocks. Transgene products are generally expressed at high levels *in vivo* and broad immune responses are induced including antigen-specific T cells and pathogen-specific antibodies. These characteristics notwithstanding, viral vectors are not a panacea. Pre-existing immunity to the vector can block transduction, concerns over vector pathogenicity are always present, and in some cases large-scale manufacturing is challenging. Viral vectors have undergone extensive preclinical assessment for a wide spectrum of diseases and have been tested in numerous clinical trials and each viral vector has its own advantages, limitations and range of applications [11,12]. There is no recombinant virus vector vaccine licensed in humans, although there are several veterinary viral vector vaccines [13,14].

The coupling of rDNA technology with only the components of a pathogen necessary to mount a protective immune response has driven vaccine development in recent decades. Subunit vaccinology was a starting point for continued technological development to provide more direct and focused immune responses that have enabled delivery of peptides, epitopes and even antibodies.

## 4. The Latest Renaissance in Vaccine Development

Despite decades of efforts and investigation, satisfactory vaccines have not yet been developed against several of the most life-threatening infections, including tuberculosis, malaria and HIV-1, which claim the lives of millions of people worldwide each year. In recent years, new technologies have emerged such as reverse vaccinology, structural vaccinology and immunoprophylaxis, which have the potential to revolutionize the vaccine field. These strategies are more complex, however they allow simpler antigen/antibody presentation in the vaccines being developed. This gives rise to the targeting of increasingly specific immune responses and strips away unessential, non-neutralizing epitopes/structures. These novel technologies represent the most valuable tools currently being applied in vaccinology and for addressing the medical needs of this century.

### 4.1. Reverse Vaccinology

The sequencing of the first bacterium genome in 1995 [15] ushered vaccine development into a new era. Suddenly, all proteins encoded by a pathogen were discernible and it became possible to identify vaccine candidates without using conventional vaccinology principles. The concept of reverse vaccinology involves screening the entire genome of a pathogen to identify genes encoding proteins with the attributes of good vaccine targets (*i.e*., surface-exposed, secreted, highly conserved among strains). Ideal candidates are selected, expressed and used to immunize mice for evaluation of immunogenicity and protection based on the analysis of antisera [16,17]. Reverse vaccinology allows for identification of a broad spectrum of vaccine candidates independent of abundance and immunogenicity during infection or ability to be cultivated in the laboratory. One of the limitations of the reverse vaccinology approach is the inability to identify nonprotein antigens such as polysaccharides, a component of many successful vaccines.

The first successful application of reverse vaccinology was for Group B meningococcus (MenB). This bacterium had been refractory to vaccine development because its capsular polysaccharide is identical to a human self-antigen and the bacterial surface proteins are extremely variable. Using reverse vaccinology, fragments of RNA were screened by computer analysis while the MenB nucleotide genome sequence was being determined. Immunoinformatics uses mathematical and computational approaches to develop algorithms to predict T-cell and B-cell immune epitopes, cellular localization of proteins (surface-exposed, secreted) by screening multiple genome sequences. Six hundred novel genes were predicted to code for surface-exposed or secreted proteins. These were cloned and expressed in *E. coli* as fusions to either glutathione transferase or a histidine tag. Of these fusion proteins, 350 were successfully expressed, purified and used to immunize mice [18,19]. The sera obtained was used to confirm surface exposure of the proteins by ELISA and FACS analysis and for the ability to induce complement-mediated *in vitro* killing of bacteria, a test correlating with vaccine efficacy in humans. Within 18 months, while the nucleotide sequence was still being finalized, 91 novel surface-exposed proteins were discovered and 28 of these were shown to induce bactericidal antibodies, a known correlate of protection against MenB disease [20]. Discovered antigens that induced the best and broadest bactericidal activity were selected and inserted into prototype vaccines that were able to induce protective immunity against most MenB strains in mice [21]. After further analysis, a final four-component MenB (4CMenB) vaccine was formulated [16].

Prior to the emergence of reverse vaccinology, only 12 surface antigens of meningococcus had been described in the literature, with four or five having bactericidal activity [18,22]. With reverse vaccinology, 91 novel surface-exposed proteins were discovered with 28 capable of inducing bactericidal activity, representing more than quarter of all novel surface-exposed proteins. The concurrent use of genomics, bioinformatics, proteomics and protein arrays can significantly accelerate identification of vaccine targets and the subsequent vaccine development process. The success of the MenB vaccine has encouraged application of reverse vaccinology to a myriad of other pathogens (both bacterial and viral), including group B streptococcus where analysis of eight genomes led to expression of 312 surface proteins and the development of a four-protein vaccine that protects against all serotypes [23].

### 4.2. Structural Vaccinology

Structural vaccinology involves rational engineering of immunogens using a combination of immunology, structural biology and bioinformatics knowledge. Antigenic epitopes are identified based on the protein amino acid sequences and the resulting secondary and tertiary structures [24,25,26,27,28]. The principles governing structural vaccinology are rooted in the observation that an efficacious immune response does not require recognition of the entire antigenic protein, but that recognition of a single or multiple selected epitopes may be sufficient to induce protective immunity. There are two distinct components of structural vaccinology; (1) conformational stabilization based on native three-dimensional structure and (2) targeting specific epitopes known to be neutralizing and protective using scaffolds.

#### 4.2.1. Conformational Stabilization

Conformational stabilization of an antigen known to elicit a protective immune response is an essential element of structural vaccinology. Desired antigens can be represented by a specific conformation of a protein that has multiple conformations (*i.e*., RSV F) or epitopes that are revealed only when proteins are in complex (*i.e*., cytomegalovirus/herpesvirus gH/gL). These antigens, when stabilized, allow a focused immune response directed towards epitopes that yield a protective neutralizing response.

The fusion protein of respiratory syncytial virus (RSV F) is a major target of structure-based vaccine design. The F glycoprotein adopts two conformations: pre-fusion and post-fusion, both are recognized by neutralizing antibodies [29]. The pre-fusion F is a metastable structure that readily rearranges into the lower energy post-fusion state [30]. There are large structural differences between the lollipop-shaped pre-fusion F trimer and the crutch-shaped post-fusion F trimer [31,32,33]. Absorption of human sera with post-fusion F fails to remove most of the F-specific neutralizing activity suggesting there are neutralizing antigenic sites unique to the pre-fusion form [34]. Elucidation of the crystal structure of the pre-fusion state of the RSV F in complex with D25 neutralizing antibody [32] paved the way for structure-based design of a stable pre-fusion F antigen with superior immunogenicity when compared to the post-fusion antigen [35]. The major target of RSV-neutralizing antibodies elicited by natural infection was found to reside on the pre-fusion conformation of RSV F at antigenic site Ø [32,34]. Antibodies, such as 5C4 [32], AM22 (Patent Application 12/600,950) and D25 (Patent Application 12/898,325), are substantially more potent than palivizumab (Synagis®, MedImmune, Inc, Gaithersburg, MD, USA), which targets antigenic site II on RSV F. A recent study demonstrated that a modified uncleaved RSV F_ecto_ (containing mutations to the furin cleavage sites and fusion peptide region) monomer binds not only key RSV-neutralizing epitopes shared between the pre-fusion and post-fusion conformations, but also the pre-fusion specific monoclonal antibody D25 and human neutralizing immunoglobulins that do not bind to the post-fusion F [36]. The use of structural information to identify neutralizing epitopes may enable construction of a vaccine with a more robust, focused immune response. 

In another example, two surface glycoproteins, gH and gL, are highly conserved among herpesviruses and represent a major target of virus-neutralizing antibodies [37]. These glycoproteins, gH and gL (as a complex), have important roles in epithelial cell entry [38,39] and fusogenic activity with gB [40,41,42,43,44,45,46,47]. Crystal structures of the gH-gL complex showed gH intimately associated with gL [40,48] (interaction necessary for proper protein folding) and often form larger complexes, gH-gL-gO and gH-gL-UL128-131 [38,39,49,50]. Analysis of hCMV seropositive sera found antibodies specific for the gH-gL-UL128-131 complex to be significantly more potent in neutralizing hCMV infections in epithelial cells than the more abundant gB [51,52,53], the canonical target antigen for vaccine development. Using this information, polycistronic alphavirus replicon particles (VRPs) that express either hCMV gH-gL or hCMV gH-gL-UL128–131 complexes in infected cells were constructed and validated [54,55]. VRPs expressing gH-gL elicited stronger and qualitatively different neutralizing antibodies than those elicited by VRPs encoding gB, VRPs encoding gH alone or VRPs encoding gL alone [54]. gH-gL VRP induced antibodies were complement-independent and broadly cross-neutralizing [54]. VRPs expressing the pentameric complex (gH-gL-UL128–131) elicited responses neutralizing infection of epithelial cells three- to seven-fold more potently than VRP expressing gH-gL in mice, as evaluated by *in vitro* neutralization assays [55].

These findings underscore the role protein/complex conformation plays in presentation of epitopes necessary to elicit appropriate immune responses (*i.e*., protection). In some cases, stabilization of a protein complex or other structure to exhibit a neutralizing epitope may not be possible. One can imagine presenting solely the epitope recognized by the neutralizing antibody on a scaffold or by some other method that stabilizes the epitope.

#### 4.2.2. Targeting Specific Epitopes Using Scaffolds

The contacts between an antigen and neutralizing antibody define a structural epitope. Methods have been developed to transplant epitopes to scaffold proteins for structural stabilization and to design minimized antigens that retain one or more key epitopes while eliminating other potentially distracting or unnecessary features [56,57,58,59,60,61]. This new class of antigens is called epitope-scaffolds. Epitope-scaffolds provide structural mimics of neutralizing determinants that are grafted into heterologous protein scaffolds that support three-dimensional structure and provide conformational stabilization [62]. Epitope-focused vaccine design is a conceptually appealing but relatively unproven method.

Early examples of epitope-scaffolds were used to battle HIV-1 infection. Epitopes are generally transplanted to unrelated scaffold proteins for conformational stabilization. Several groups implemented this strategy using computational design to create epitope-scaffolds presenting the HIV-1 gp41 MPER (membrane-proximal external region) epitopes 2F5 and 4E10 [56,58,63]. The epitope-scaffolds bound with high affinity to the cognate antibody and epitope-scaffold structures recalled precise epitope structural mimicry. Precise mimicry is determined, in part, by crystal structure of unliganded epitope-scaffolds and antibody-bound epitope-scaffolds, binding affinity to antibody and analysis of side-chains. It was demonstrated that epitope-scaffolds bound antibody with significantly higher affinity than corresponding epitope peptide alone [58,63]. The HIV-1 epitope-scaffolds could induce epitope structure-specific antibodies from small animals, however, sera from animals vaccinated with 2F5 or 4E10 epitope-scaffolds did not neutralize HIV-1 as assessed by *in vitro* neutralization assays [56,58]. The lack of neutralization by these epitope-scaffolds may be due to an incomplete epitope or missing portions of the epitope essential to induce neutralizing antibodies. A number of other groups have studied expression of the nominal 2F5-epitope sequence (ELDKWAS) in a number of scaffold systems; (1) a variable loop of HIV-1 gp120 envelope glycoprotein, (2) a surface loop of human rhinovirus and (3) a surface loop of bovine papilloma virus [64,65,66,67]. All these systems elicited antibodies targeted to the ELDKWAS epitope and several induced weak neutralizing antibodies [64,65,67]. The newest computational protocol, MultiGraft Interface, transplants epitopes and designs additional scaffold features outside the epitope to enhance antibody-binding specificity and potentially influence the specificity of elicited antibodies. This protocol was used to engineer novel epitope-scaffolds that display the HIV-1 neutralizing antibody 2F5 epitope and interact with the functionally important CDR H3 antibody loop. The resulting epitope-scaffold bound 2F5 with subnanomolar affinity [68]. Epitope-scaffold technology represents a successful example of rational protein backbone engineering and protein-protein interface design and could prove useful in the field of HIV-1 vaccine design.

This technology has also been applied to RSV. The clinical use of the monoclonal antibody, palivizumab (Synagis®, MedImmune, Inc.), directed against a single antigenic site (residues 255–275, 13 residues in two α-helices) on the fusion (F) glycoprotein, is sufficient to prevent severe disease caused by RSV [69,70]. Peptides corresponding to this region bind to palivizumab-like antibodies but fail to elicit neutralizing antibodies when injected in mice [71]. These results suggest that the free peptide fails to mimic the correct conformation of the epitope. McLellan *et al*. applied and extended the methodology used with the HIV-1 epitopes 2F5 and 4E10, to the RSV motavizumab epitope (recognizes same antigenic site as the palivizumab epitope), with a computational method to identify scaffold proteins able to support a discontinuous epitope structure [72]. The identified epitope-scaffolds improved upon peptide immunogens by maintaining the epitope in a conformation approximating its antibody-bound state [72]. The epitope-scaffolds failed to elicit detectable RSV-neutralizing activity in sera of immunized mice despite increased conformational stability and ability to bind RSV F, most likely due to the low titers of epitope-specific antibodies [72]. Building on the response with the motavizumab epitope-scaffold, Correia *et al.* designed scaffold proteins for RSV F motavizumab epitope with full backbone flexibility, and favorable biophysical and structural properties, that mimicked the viral epitope structure [73]. Immunization with these epitope-scaffolds induced potent neutralizing antibodies in the majority of macaques. Monoclonal antibodies isolated from one vaccinated macaque had unusually high affinity for the eliciting antigen [73].

Recent results have decisively established the potential of epitope-focused and scaffold-based vaccine design for viruses that have previously resisted vaccine development by more traditional methods. As our understanding of detailed three-dimensional structure, domain organization and dynamics of surface proteins of pathogens improves, it will offer molecular targets that can guide the design of effective vaccines and better immunogens through stabilization of native conformations or combining, exposing, and/or improving the immunogenicity of epitopes [24,62,74].

An important consideration with this technology is that focus on a single epitope could potentially fail due to mutation of that epitope in the pathogen. Epitope selection should focus primarily on conserved epitopes, across many strains. Conserved epitopes are unlikely to be subject to the same mutational pressures as unconserved or highly variable regions. We focused on studies involving conserved epitopes. In situations where stabilization of a protein or epitope eliciting neutralizing antibodies is not possible, directly injecting a known neutralizing antibody that protects against infection/disease may circumvent that issue.

### 4.3. Immunoprophylaxis by Gene Transfer

Vectored delivery of antibodies directed against infectious pathogens allows protection without requiring the mounting of an immune response (immunoprophylaxis), which is traditionally generated by active immunization. There are two approaches for use of isolated neutralizing antibodies. The first is passive immunization of neutralizing antibodies that protect against infection. However, often multiple injections of antibodies are necessary due to antibody half-life, which is neither cost effective nor practical as a vaccine strategy. Not discussed in this section, but of equal importance, are efforts to improve antibody production, formulation and half-life. The second is to isolate the neutralizing antibody gene and use gene transfer technology to endow a target host with the gene. With vector-mediated gene transfer, the antibody gene is delivered to the host using recombinant adeno-associated virus (rAAV) vectors, resulting in long-term endogenous antibody expression from the injected muscle conferring protective immunity. The rAAV gene transfer vectors are devoid of the endogenous *rep* and *cap* genes and consist of the antibody gene expression cassette flanked by the AAV ITRs (inverted terminal repeats), the only part of the AAV genome present in the rAAV vector. The ITRs (145 bp each) are necessary for rAAV vector genome replication and packaging. The rAAV vectors (with heavy- and light-chain antibody genes incorporated into a single vector, either in a two-promoter system or a single promoter for expression with heavy and light chain being separated by a foot-and-mouth disease virus 2A peptide) have been shown to transduce muscle *in vivo* with high efficiency and direct the long-term expression of a variety of transgenes [75,76,77,78,79,80,81]. Multiple AAV serotypes have been identified with varying transduction efficiencies in different tissues, offering flexibility for gene transfer targets [82]. This strategy is promising in several respects: (1) gene transfer bypasses the adaptive immune response, (2) the antibody transgene(s) of interest can be “pre-selected” and (3) multiple steps in a pathogen lifecycle can be targeted.

The concept of rAAV-mediated antibody gene transfer was tested in animals by using one of the first broadly neutralizing HIV-1 antibodies isolated, IgG1b12. The resulting rAAV vector was injected into the muscles of immunodeficient mice. IgG1b12 was expressed in mouse muscle and biologically active antibody (determined by an HIV-1 neutralization assay against IgG1b12-sensitive/resistant viruses) was found in sera for over six months [81]. This study provided the first evidence that rAAV vectors (1) transferred antibody genes to muscle, (2) myofibers produced antibodies, (3) antibodies were distributed into circulation and (4) antibodies were biologically active.

The next objective was to test the gene transfer concept in macaques in a challenge study. In pilot experiments using the rAAV-IgG1b12 vector, macaques developed antibody responses to the human-derived transgene that effectively shut down expression. To avoid this, a native macaque SIV gp120-specific Fab molecule, derived directly from an SIV-infected macaque, was used as an immunoadhesin [83]. Immunoadhesins are chimeric, antibody-like molecules combining the functional domain of a binding protein like a single chain variable fragment or CD4 extracellular domains 1 and 2 with an immunoglobulin constant domain [84]. Using this approach, macaques were able to generate long lasting neutralizing activity in serum and were completely protected against intravenous challenge with virulent SIV [80]. Longitudinal studies of the protected macaques (now over 6 years post-injection) showed immunoadhesin levels to be stable for the past four years with macaques remaining negative for SIV infection [85]. Another group used rAAV vector-mediated gene transfer expression/challenge studies to express native, full antibodies of 2G12, IgG1b12, 2F5, 4E10 and VRC01 [86]. Following intramuscular rAAV injection in mice, antibody expression levels greater than 100 μg/mL were observed for at least 12 months and in a humanized mouse model, rAAV vectors provided protection from HIV challenge with antibody serum levels as low as 8.3 μg/mL (VRC01) [86].

A novel application of immunoprophylaxis by gene transfer involved the fusion of the immunoadhesin form of CD4-Ig with a small CCR5-mimetic sulfopeptide at the carboxy-terminus (eCD4-Ig), targeting two of the most conserved epitopes of the HIV-1 envelope [87]. eCD4-Ig binds avidly and cooperatively to the HIV-1 envelope glycoprotein and is more potent than the best broadly neutralizing antibodies. Rhesus macaques inoculated with AAV vector stably expressed 17–77 μg/mL of fully functional rhesus eCD4-Ig for more than 40 weeks. These macaques were protected from multiple infectious challenges, of increasing dose, with SHIV-AD8.

In moving this technology into humans, the first clinical trial using rAAV vector-mediated antibody gene transfer started in January 2014 as a result of a collaboration between The Children’s Hospital of Philadelphia, the International AIDS Vaccine Initiative and the Division of AIDS. The rAAV vector expresses the PG9 full antibody (a potent glycan dependent broadly neutralizing antibody). The current PG9 study will provide important information about vector safety, antibody concentration, duration of expression and neutralization capacity in humans although future human trials would utilize even more potent antibodies, such as PGT121 [88,89] or PGDM1400 [90]. Ultimately, antibody gene transfer vectors targeting all steps in HIV-1 entry will create a multilayered blockade against HIV infection.

Besides HIV-1, the rAAV-mediated antibody gene transfer system can be applied to many other pathogens—including influenza and respiratory diseases. Limberis *et al.* engineered an AAV vector, serotype 9, to express a modified version of the previously isolated broadly neutralizing influenza monoclonal antibody, FI6 [91]. AAV9, a natural variant of AAV, efficiently transduces proximal and distal airway epithelial cells in mice after nasal delivery [92], the initial site of infection for respiratory viruses, such as influenza and RSV. Localized, durable and high-level expression of the broadly neutralizing influenza antibody, FI6, was achieved in the nasal epithelia of mice and ferrets with AAV9 vectors. Treatment provided almost complete protection against a wide range of clinical pandemic influenza isolates in both animal species [91]. In an additional study, young, old and immunodeficient mice treated intranasally with AAV9 expressing FI6 were protected and exhibited no signs of disease following intranasal challenge with the mouse-adapted H1N1 influenza strain [93]. Balazs *et al*. showed that a single intramuscular injection of AAV encoding a broadly neutralizing influenza antibody (either F10 or CR6261) was capable of protecting young, old and immunodeficient mice against diverse influenza strains (all H1, H2 and H5 influenza strains tested) [94]. These studies demonstrated the broad spectrum of efficacy in animal models using antibody gene transfer directed against influenza and serve as a platform for the prevention of natural respiratory infection for which a protective antibody has been identified.

Immunoprophylaxis by gene transfer has primarily involved the use of the AAV vector, however it does not preclude other vectors from being feasible. AAV has been shown to be a popular gene delivery vehicle for use in clinical studies for treatment of disease such as alpha-1-antitrypsin deficiency, cystic fibrosis, hemophilia B, Parkinson’s and muscular dystrophy, among others, and has an established record of high-efficiency gene transfer in a variety of model systems [78,79,95]. Despite this approach holding great promise, there are several factors that may limit its effectiveness and use as a vaccine; (1) pre-existing immunity to AAV, (2) immunogenicity of the selected antibody, (3) anti-antibody responses and (4) potential for off-target binding by the antibody. (1) Approximately 80% of the human population is seropositive for AAV with antibodies directed towards AAV1 and AAV2 being the most prevalent. AAV does not cause any disease or other pathological condition in humans despite early and repeated exposures to a number of AAV serotypes [96,97,98]. The presence of pre-existing neutralizing antibodies, even with low titers, can have a negative impact on vector transduction [99,100,101,102,103]. Furthermore, neutralizing antibodies may prevent repeated administrations [100], which would impede rAAV delivery of potentially more potent antibodies at a later time. Potential solutions to the AAV neutralization conundrum involve using rare AAV capsids, or capsids that have been reengineered to remove or alter neutralization epitopes [82]. (2) Several factors contribute to the immunogenicity of an antibody including structure, dose and recipient’s genetic background. More than 20 monoclonal antibodies have been used as therapeutics [104] and all have exhibited some level of immunogenicity [105,106]. (3) In non-human primate studies [80], the appearance of anti-antibody responses resulted in loss of transgene expression with no adverse events being observed. (4) Another potential concern is the risk of an antibody binding to an off-target host protein causing an adverse event, which has been suggested for HIV antibodies 2F5 and 4E10 [107]. One way to predict this occurrence is to do tissue-binding studies with purified proteins or expression in mouse models [108].

## 5. Challenges for Modern Vaccine Development

The sections above outline the history of successful vaccines and the technology/approach that resulted in those vaccines. We also described some of the current technologies being applied to vaccine development for pathogens that have resisted more traditional/conservative vaccine approaches. For each of the emerging technologies, we gave examples of how these technologies were being utilized for vaccine development, in animal studies and in some cases, clinical testing in humans. There are many technologies and approaches not described in this review; *i.e*., deceptive imprinting, immune refocusing, RNA, nanotechnologies, among others.

Many challenges to vaccine development exist. The type of immune response elicited by a vaccine (*i.e*., humoral *vs*. cellular responses), the strength (*i.e*., does it protect against any level of infection, reduce severe infection) and longevity of (*i.e*., single immunization or are boosters necessary) that response could significantly impede vaccine development. Vaccine development can be further hindered by how the immune response is generated (*i.e*., protein, epitope, antibody) and delivered (*i.e*., viral vectors, adjuvants, formulated). Once a vaccine is developed through study in animal models, there can be challenges with scale up for mass production (*i.e*., use in human trials), dosing, route of administration (*i.e*., intramuscular, intranasal, oral) and effectively protecting diverse target populations (*i.e*., infants, immunocompromised individuals, elderly). Each of these facets introduces additional elements that need to be studied, understood and addressed when developing a vaccine.

Current technologies are primarily focused on improving the quality of the immune response via rational design of the antigen through stabilizing complex structure(s) and identifying neutralizing epitopes. High levels of variability in antigenic proteins can pose a unique barrier to vaccine development—how to target a protein or epitope subject to selective pressures? Often the most abundant antibodies in seropositive sera are not the most potent or effective at neutralizing infection, as is the case with CMV, where the majority of antibodies target gB but the most potent are directed towards the gH/gL/UL128–131 complex. Additionally, the limited capacity of the immune system to develop potent, sustained antibody responses at the extremes of age or undetectable antibody responses to infections and immunizations can hinder the viability of a vaccine. The role of waning maternal antibodies and an immature immune system in infants can complicate the immune response elicited by a vaccine whereas the elderly show a significant decline in capacity to induce protective titers. The point at which a vaccine is determined to be effective depends on the end point measurement. Does a vaccine need to prevent all infection or only prevent severe infection or reduce hospitalization/health care costs? It depends on the pathogen.

These challenges, in addition to many others, come into play when designing and evaluating a potential vaccine. Despite the challenges, vaccination represents a successful means to prevent infectious diseases and improve public health.

## 6. Conclusions

Vaccine-preventable illnesses continue to place a heavy burden on the human population and health care systems. For maximal, affordable and sustainable gains in global health, new or improved vaccines are needed for several major pathogens including HIV-1, HCV, malaria, tuberculosis, influenza, dengue virus and RSV. Although most current vaccines are based on either live-attenuated or whole-inactivated viruses, these approaches have not worked or are considered unsafe for some of the pathogens awaiting successful vaccine development [109]. The emergence of reverse vaccinology, structural vaccinology and immunoprophylaxis represent valuable tools. These tools, combined with our growing understanding of human immunology, provide powerful strategies for the rational design of engineered vaccines bearing multiple antigenic epitopes offering the opportunity of developing broadly effective immunity [25,26,110].
